# Infusion-related thrombogenesis by liver-derived mesenchymal stem cells controlled by anticoagulant drugs in 11 patients with liver-based metabolic disorders

**DOI:** 10.1186/s13287-020-1572-7

**Published:** 2020-02-07

**Authors:** Louise C. F. Coppin, Françoise Smets, Jérome Ambroise, Etienne E. M. Sokal, Xavier Stéphenne

**Affiliations:** 1 Service de Gastro-Entérologie et Hépatologie Pédiatrique, Cliniques Universitaires Saint-Luc, Université Catholique de Louvain, Av Hippocrate 10, B-1200 Brussels, Belgium; 2 Institut de Recherche Expérimentale et Clinique, Université Catholique de Louvain, Brussels, Belgium

**Keywords:** Mesenchymal stem cells, Cell therapy, Blood coagulation, Clinical trial, Liver diseases

## Abstract

**Background:**

Mesenchymal stem cell (MSC) transplantation is a fast-developing therapy in regenerative medicine. However, some concerns have been raised regarding their safety and the infusion-related pro-coagulant activity. The aim of this study is to analyze the induced thrombogenic risk and the safety of adding anticoagulants during intraportal infusions of liver-derived MSCs (HepaStem), in patients with Crigler-Najjar (CN) and urea cycle disorders (UCD).

**Methods:**

Eleven patients (6 CN and 5 UCD patients) were included in this partially randomized phase 1/2 study. Three cell doses of HepaStem were investigated: low (12.5 × 10^6^ cells/kg), intermediate (50 × 10^6^ cells/kg), and high doses (200 × 10^6^ cells/kg). A combination of anticoagulants, heparin (10 I.U./5 × 10^6^cells), and bivalirudin (1.75 mg/kg/h) were added during cell infusions. The infusion-related thrombogenic risk and anticoagulation were evaluated by clinical monitoring, blood sampling (platelet and D-dimer levels, activated clotting time, etc.) and liver Doppler ultrasound. Mixed effects linear regression models were used to assess statistically significant differences.

**Results:**

One patient presented a thrombogenic event such as a partial portal vein thrombus after 6 infusions. Minor adverse effects such as petechiae, epistaxis, and cutaneous hemorrhage at the site of catheter placement were observed in four patients. A significant decrease in platelet and increase in D-dimer levels were observed at the end of the infusion cycle, normalizing spontaneously after 7 days. No significant and clinically relevant increase in portal vein pressure could be observed once the infusion cycle was completed.

**Conclusions:**

The safety- and the infusion-related pro-coagulant activity remains a concern in MSC transplantation. In our study, a combination of heparin and bivalirudin was added to prevent the thrombogenic risk induced by HepaStem infusions in 11 patients. A significant decrease in platelet and increase in D-dimer levels were observed, suggesting the activation of coagulation in these patients; however, this was spontaneously reversible in time. We can conclude that adding this combination of anticoagulants is safe and limits infusion-related thrombogenesis to subclinical signs in most of the patients.

**Trial registration:**

ClinicalTrials.gov identifier: NCT01765283—January 10, 2013

## Background

Orthotopic liver transplantation (OLT) is still the current treatment of choice for patients presenting end-stage liver disease or liver-based inherited metabolic diseases, when conservative treatment cannot maintain a sufficient liver function or quality of life. However, due to organ shortage, the growing demand for organs and the substantial risks of morbidity and mortality linked to immunosuppression and surgery, other treatments, such as cell-based therapy, are being developed. In metabolic patients, the aim is to provide the missing specific liver functions and prevent the metabolic decompensation and related side effects. Hepatocyte transplantation can be an alternative treatment for liver-based metabolic conditions [1–7]. However, this treatment is limited due to the inability of hepatocytes to proliferate in vitro, due to the metabolic damages induced by cryopreservation and to organ shortage as well [8–10]. Other cell sources, such as mesenchymal stem cells (MSCs), are currently under evaluation in numerous clinical trials. Unlike hepatocytes, MSCs can be isolated and expanded efficiently in vitro. They are undifferentiated cells that can differentiate after transplantation, under the influence of local environmental signals, into cells expressing the appropriate phenotype [11]. Patients presenting urea cycle disorders (UCDs) and Crigler-Najjar (CN) syndrome are good candidates for MSC transplantation, because their current treatments are heavy, only supportive, and impose a real burden on the patients and their family.

Previous studies [12] showed that liver-derived MSCs or HepaStem, like other MSCs [13–16], express a procoagulant activity (PCA) that cannot be inhibited using one anticoagulant drug individually. Due to the overexpression of tissue factor (TF), MSCs in contact with blood activate coagulation and induce a thrombogenic risk in the transplanted patients. Several infusion-related thrombi have been reported in literature [17–22]. Bennet et al. [23] were the first to describe a thrombo-inflammatory reaction when pancreatic islets, expressing TF, were in contact with blood, which they called instant blood-mediated inflammatory reaction (IBMIR). IBMIR has been described as a dual activation of the coagulation and the complement pathway by islets or cells bearing TF. Transplanted cells are rapidly encapsulated in a blood clot, with infiltration by activated polymorphonuclears (PMNs), causing early cell destruction, leading to poor cell engraftment [12, 24–27].

Nonetheless, the procoagulant activity of HepaStem can be limited, in in vitro models, using a combination of anticoagulants, including an antithrombin activator (heparin) and thrombin inhibitor (bivalirudin) [12]. The objective of this study is to analyze the thrombogenic risk induced by HepaStem infusion and the safety of the use of this anticoagulant cocktail in CN and UCD patients.

## Methods

### Health authorities and ethical review

Ethical approval to report this case series was obtained from Belgian regulatory authorities and the ethical committee (2011/04OCT/388). All procedures in this study were conducted in accordance with the European Medicines Agency’s (EMA) pediatric committee (EudraCT number: 2011-004074-28) and conducted according to the 2000 revised principles of the Declaration of Helsinki (ClinicalTrials.gov identifier: NCT01765283). Written informed consent was obtained from the patient or a legally authorized representative(s) for anonymized patient information to participate in this study.

### Study population

The data we developed hereafter are part of a multicentric phase 1–2 clinical trial which described the therapeutic effects of the cell therapy [28]. Included patients had a confirmed diagnosis of Crigler-Najjar (CN) syndrome (I or II) or urea cycle disorder (UCD) (ornithine transcarbamylase (OTC) deficiency or arginase deficiency) by genetic mutation analysis. General inclusion criteria included patient and/or legal representative providing written informed consent, negative pregnancy test for a female subject with childbearing potential, and patency of the portal vein and branches. For CN patients, specific inclusion criteria included the diagnosis of CN poorly nonresponsive to phenobarbital treatment. Specific inclusion criteria for UCD patients included a disease of such severity (e.g., poor protein tolerance and recurrent hyperammonemic crises despite maximal conservative metabolic treatment) to warrant liver transplantation or alternatives. Major exclusion criteria included acute liver failure, clinical or radiological evidence of liver fibrosis or cirrhosis, known medical or family history of coagulopathy, and the presence of a thrombosis of the portal vein or persisting impairment of anterograde portal blood flow.

We included 11 patients (6 CN and 5 UCD patients) in this prospective, open-label, partially randomized, dose-escalation phase 1/2 study (see Table 1, and consort flow diagram in Additional file 1) (ClinicalTrials.gov Identifier: NCT01765283). For ethical reasons, the placebo effect was not studied. Three cell doses were investigated: low (12.5 × 10 cells/kg), intermediate (50 × 10^6^ cells/kg), and high (200 × 10^6^ cells/kg) (4 × 10^9^ maximum total cell count). Cells were administered as one cycle of one or several infusions, depending on the assigned cell dose and the patient’s body weight, with a maximum of 250–500 × 10^6^ cells. Thereafter, an interval of 2–6 h was taken before reinfusion of the next cells.

**Table 1:** Population

	Number of patients
Infused patients	11
Crigler-Najjar	6
Type 1	5
Type 2	1
Urea cycle defect	5
OTC deficiency	4
Arginase deficiency	1
Sex	
Female	7
Male	4
Age at infusion	0.9–16.25 year(s)
Patients per cell dosage	
12.5 × 10^6^/kg	4
50 × 10^6^/kg	2
200 × 10^6^/kg	5
Total number of infusions	1–10
Total number of days of infusions	1–4
Completed infusion cycles	7

### Cell preparations

Liver-derived mesenchymal stem cells or HepaStem were obtained from healthy human donor livers (*n* = 3) as previously described [29]. No organs from executed prisoners were used. Cells were purified, characterized, and expanded in vitro for five successive passages, then harvested, cryopreserved in CryoStor-10 (10% dimethyl sulfoxide (DMSO)), and stored in liquid nitrogen. Before use, the cells were thawed and washed in human albumin solution 5% to remove DMSO. Then, they were conditioned in bags of 50 ml in an infusion solution (human albumin 5%, heparin 10 I.U./ml (Heparin Leo®, Leo Pharma, Ballerup, Denmark)) at a concentration of 5 × 10^6^ cells per milliliter. Final heparin concentration was 10 I.U. per 5 × 10^6^cells; therefore, patients received respectively between 25 and 400 I.U. of heparin/kg during the whole infusion cycle depending on the cell dose used, with a maximum of 1000 I.U. of heparin per infusion. The infused cells presented a viability of 60–80% at the trypan blue test. Each patient received cells from the same donor.

### Infusion protocol

Cells were infused intravenously through a percutaneous transhepatic catheter inserted into the portal vein under radio guidance or via catheter inserted surgically in an affluent of the portal vein. Before each infusion, an ultrasound was performed to assure the catheter was in place and permeable. Surveillance of the portal-vein pressure was maintained throughout the entire infusion procedure and after the cycle of infusions. The flow rate of infusion was kept at 0.5–2 ml/min.

### Concomitant therapy

Before and during the infusion period, a prophylactic anticoagulation treatment, bivalirudin (Angiox®, The Medicines Company, Abingdon, UK), was concomitantly administered to all the patients. The same treatment protocol was used for percutaneous coronary interventions. Bivalirudin was started 15 min ahead of the cell infusion to provide a loading dose. Through the infusion, we administered a dose of 1.75 mg/kg/h that was reduced to 0.25 mg/kg/h at the end of the cell infusion, until re-start of the next cell infusion, for a maximum of 2 h (if next infusion was scheduled). Immunosuppressive treatment was given to each patient through the entire study duration. A single bolus of Solumedrol (Solu-medrol®, Pfizer, New York City, NY, USA) (2 mg/kg) a day was given before the infusion. Basiliximab (Simulect®, Novartis Pharma, Basel, Switzerland) was given on day 0 (day of the first infusion) and day 4 (5 mg/day for < 15 kg body weight, 10 mg for 15–35 kg, 20 mg for > 35 kg). Tacrolimus (Prograft®, Astellas Pharma, Tokyo, Japan) therapy was started at 0.3 mg/kg, with dose adjustments to reach as quickly as possible target blood levels of 10 ± 2 ng/ml. Antibiotics were administered before the placement of the catheter to prevent post-operative infections according to institutional guidelines. They also receive chemoprophylactic medication to prevent opportunistic infections (antiviral, antimicrobial) according to current recommendations of chemoprophylaxis after liver transplantation.

### Safety and monitoring of the patient

Vital signs (heart rate, blood pressure, oxygen saturation, and respiratory rate) were monitored through the entire infusion. The monitoring of the anticoagulation therapy was done with the measurement of activated clotting time (ACT) and ROTEM® delta analyzer (Pentapharm, Munich, Germany) at selected times. ACT was measured before, and every 30 min during and after the cell infusion. Cell infusion was started once ACT levels were > 200 s and kept at values between 200 and 350 s during the cell infusion. If values exceeded 350 s, bivalirudin was reduced from 1.75 to 0.25 mg/kg/h with ACT measurements every 15 min until the value(s) were between 200 and 350 s. D-dimers (normal values < 500 ng/ml) were measured after the catheter placement, prior to and after the infusions, and the morning after the cycle was completed. If the patients presented elevated values of D-dimer (> 20.000 ng/ml), an ultrasound was performed to evaluate the permeability of the catheter. Blood samples were collected before and after each cell infusion, and on days 1, 3, and 7 post-infusion once the infusion cycle was completed. The following parameters were studied: hemoglobin (normal values 11–14.5 g/dl), platelets (normal values 150–350 × 10^3^/μl), white blood cells (normal values: 4–10 × 10^3^/μL), polymorphonuclear (PMN) (normal values 5.6–17 × 10^3^/μl), lymphocytes (normal values 1.4–3.8 × 10^3^/μl), and monocytes (normal values 0.2–1.3 × 10^3^/μl). Hepatic ultrasound and Doppler, to measure the main portal flow (normal values 20–40 cm/s [30]) and control the absence of thrombi, were performed before and after each infusion, and on days 1, 3, 30, and 90 post-infusion once the infusion cycle was completed. Portal vein pressure (normal values 7–12 mmHg [31]) through a portal catheter was recorded before and every 15 min during and after the infusions until normalization.

### Statistical analysis

Mixed effects linear regression models were used to assess the impact of time on various outcomes. In each model, the “patient” random effect was included to model the inter-patient variability while the fixed “time” effect was used to assess the increase or decrease of the outcome over time. All statistical analyses were performed using R.3.4.0 and Graphpad 5.0.

## Results

### Study population

Eleven patients were treated with HepaStem infusions, of which five UCD and six CN. Two UCD and two CN patients were assigned to low dose (12.5 × 10^6^ cells/kg), one UCD and one CN to medium dose (50 × 10^6^ cells/kg), and three UCD and two CN to high dose. The total infusion cycle was administered in 7 patients over 1–4 days in 1–11 infusions (see Tables 1 and 2). The high dose could not be administered completely to four patients due to catheter displacement (*n* = 2), transfusion reaction (*n* = 1), and high D-dimer levels > 20.000 (nl < 500 ng/ml).

**Table 2:** Thrombogenic risk

	Total patients	% of the total population	12.5 × 10^6^/kg	50 × 10^6^/kg	200 × 10^6^/kg
Complication related to anticoagulation	4	36			
Hemorrhage at catheter	1	9	–	–	1
Epistaxis	2	18	1	1	–
Petechiae	1	9	–	–	1
Procoagulant events	1	9			
Portal thrombi	1	9	–	1	–
Prematurely stopped infusion cycles	4	36			
Catheter problems	2	18	–	–	2
Transfusional reaction	1	9	–		1
High D-dimer levels	1	9			1

### Safety

#### Thrombogenic risk

**Fig. 1:**
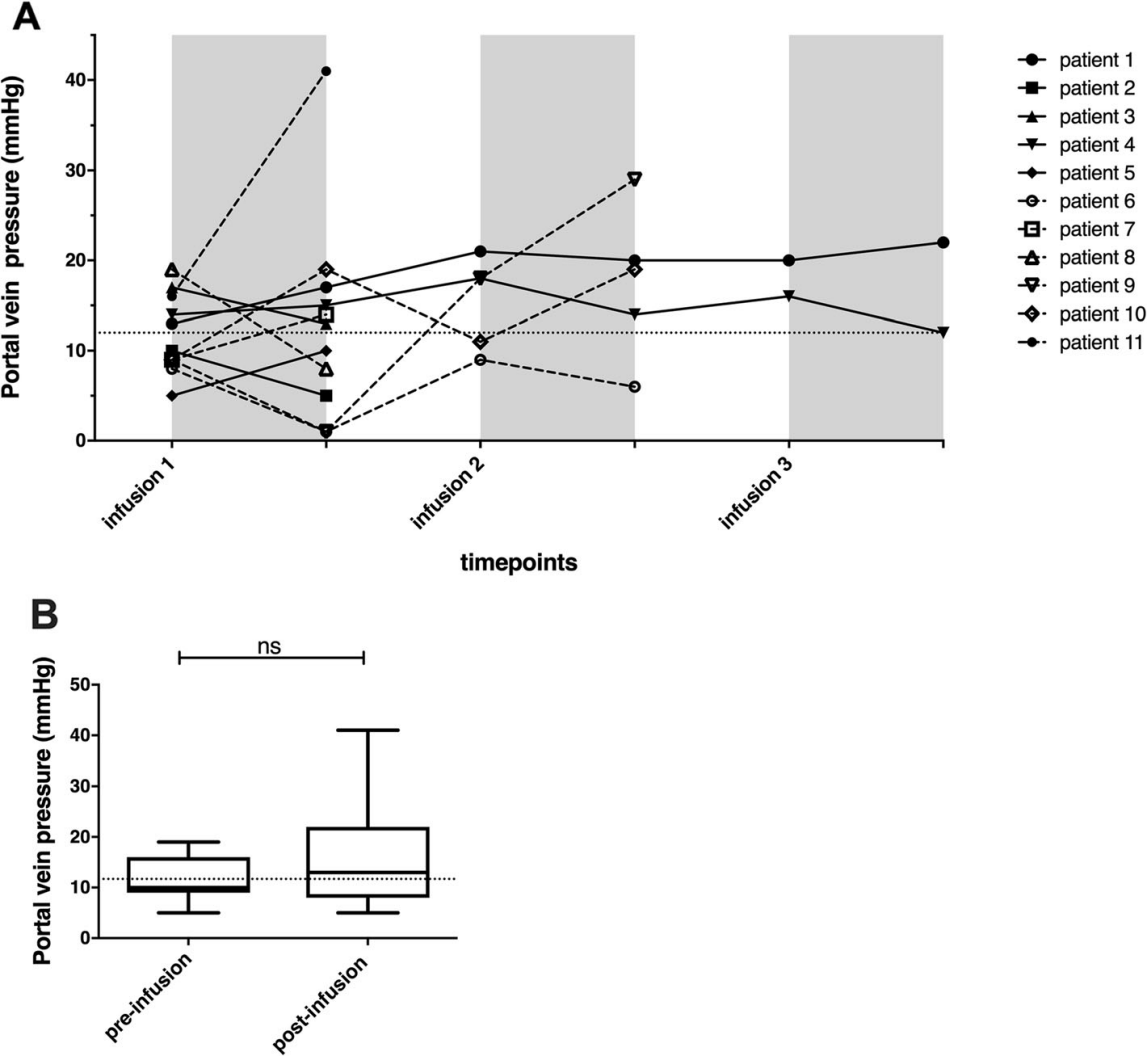
Monitoring of portal vein pressure before and after intraportal infusion of HepaStem in patients (*n* = 11). Portal vein pressure was monitored through a portal catheter before and every 15 min during and after the infusions until normalization. **a** Portal vein pressure is represented for each patient during 1, 2, or 3 days of HepaStem infusions. The horizontal dotted line is the maximum normal value. **b** 95% CI (confidence interval) whiskers representing portal vein pressure for all patients at different time points. Statistical analysis: Wilcoxon signed-rank test. The horizontal dotted line represents the mean before infusion. *p < 0.05; **p < 0.01; ***p < 0.001

**Fig. 2:**
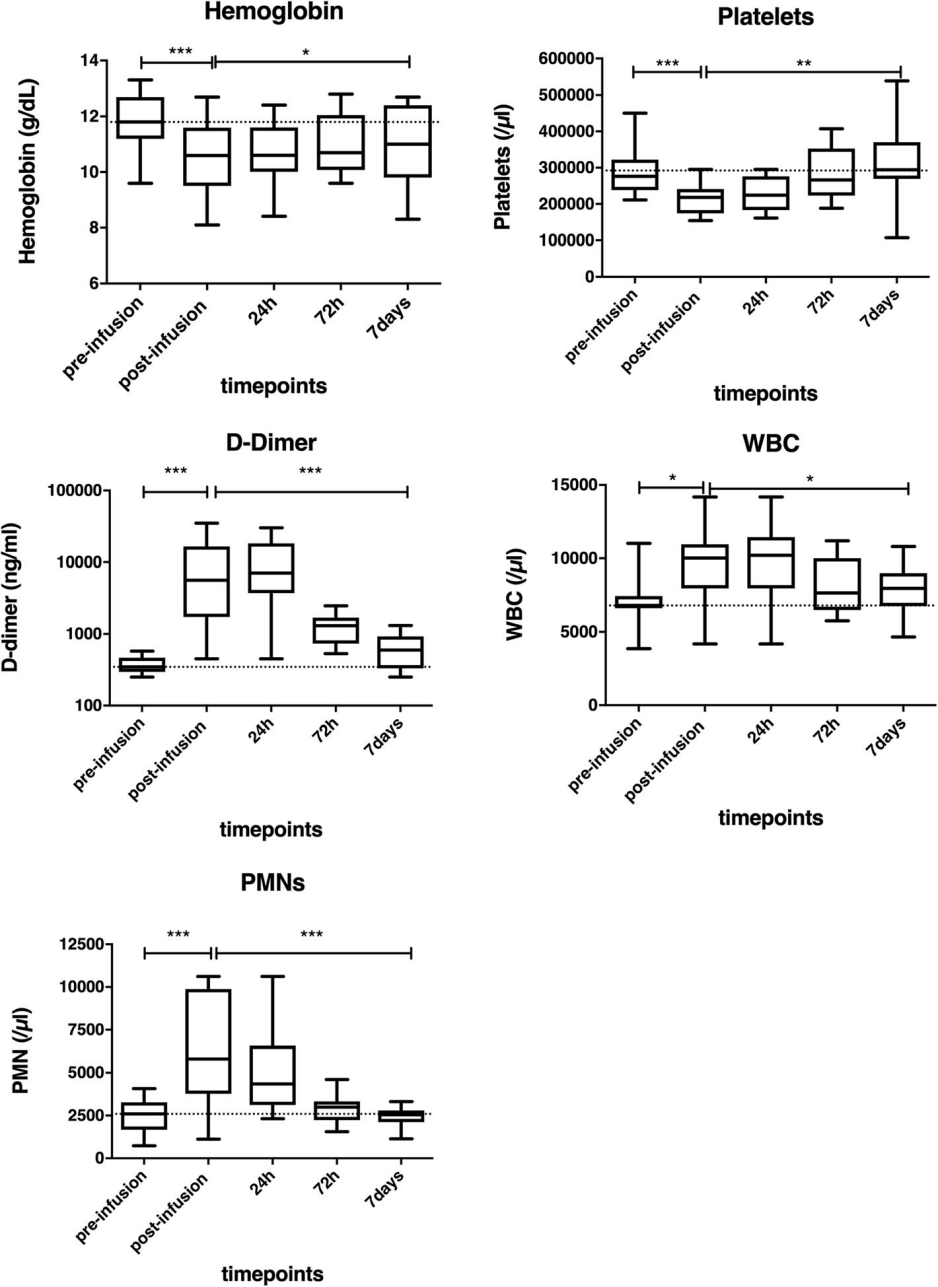
Monitoring of blood parameters before and after intraportal infusion of HepaStem in patients (*n* = 11). Blood samples were performed before, at the end, and 1, 3, and 7 days after, the infusion cycle was completed. Blood samples included the following: hemoglobin (normal values 11–14.5 g/dl), platelets (normal values 150–350 × 10^3^/μl), white blood count (normal values 4–10 × 10^3^/μL), polymorphonuclear (PMN) (normal values 5.6–17 and D-dimer levels (normal values < 500 ng/ml), and 95% CI (confidence interval) whiskers. The horizontal dotted line represents mean before infusion. *p < 0.05; **p < 0.01; ***p < 0.001

Vital signs stayed in the normal range during and after cell infusions. One OTC patient presented an intraluminal non-occlusive left portal vein thrombus the second day of infusions. Liver ultrasound showed a mild increase in portal vein pressure (22 mmHg–normal value 8–12 mmHg). This was resolved after 1 month with curative low molecular weight heparin treatment. Two other patients presented high portal vein pressure (> 25 mmHg) during cell infusion at the ultrasound. In one patient, the infusion cycle was interrupted for concomitant high D-dimer levels (> 35.000 ng/ml) (normal values < 500 ng/ml), followed by a fivefold increase in liver enzymes (AST and ALT) 3 days after the infusion cycle was interrupted. They normalized after 14 days without administration of additional anticoagulation treatment. Nevertheless, only a minor, non-significant and clinically non-relevant increase in portal vein pressure has been observed before and after infusions in the whole population of the study (see Fig. 1). Intraportal HepaStem infusion affected the following blood parameters (see Fig. 2 and Table 3).

**Table 3:** Evolution of blood parameters

		Before-after infusion			1 week after infusion		
	Normal values	mean	CI 95%	*p* value	mean/day	CI 95%	*p* value
Hemoglobin	11–14.5 g/dL	− 1.3 g/dL	0.7–1.8	0.001	0.06 g/dL	0.005–0.1	0.05
Platelets	150–350 × 10^3^/μL	− 77 × 10^3^/μL	40–110	0.001	15 × 10^3^/μL	8–22	0.01
White blood count	4–10 × 10^3^/μL	2.7 × 10^3^/μL	0.6–4.8	0.05	− 0.3 × 10^3^/μL	0.005–0.5	0.05
Polymorphonuclear	5.6–17 × 10^3^/μL	+ 150%	61–288	0.001	− 12%	8–16	0.001
Lymphocytes	1.4–3.8 × 10^3^/μL	− 1.2 × 10^3^/μL	0.1–2.4	0.05	2.2 × 10^3^/μL	1.2–3.2	0.001
Monocytes	0.2–1.3 × 10^3^/μL	0.1/μL	− 0.4–0.3	0.14	− 35/μL	14–56	0.05
D-dimer	< 500 ng/mL	+ 1400%	500–3800	0.001	− 31%	24–38	0.001
Portal vein pressure	7–12 mmHg	4.7 mmHg	− 2.5–11.9	0.26			

### Difference in blood parameters before and after the infusion and evolution each day till 7 days after the infusion by mixed effects linear regression models, results shown are means, confidence interval 95% (CI 95%), and *p* values

D-dimers increased significantly with 1442% (CI95% 508–3810%; *p* < 0.001) between the beginning and the end of the infusion cycle. After the infusions, D-dimers decreased significantly with 31% per day (CI95% 24–38%; *p* < 0.001) to return to normal levels 7 days after the whole cycle was completed. Hemoglobin (normal values 11–14.5 g/dl) decreased significantly with 1.3 g/dl (CI95% 0.7–1.8 g/dl; *p* < 0.001) after infusion, but increased again significantly with 0.06 g/dl per day (CI95% 0.005–0.1 g/dl; *p* = 0.03) during 7 days after the infusion. Platelets (normal values 150–350 × 10^3^/μl) decreased significantly with 77 × 10^3^/μl (CI95% 40–114 × 10^3^/μl; *p* < 0.001) due to the infusions, but normalized 7 days after the whole cycle was completed by increasing significantly with 15 × 10^3^/μl (CI95% 8–22 × 10^3^/μl; *p* < 0.01) daily. Total white blood counts (normal value 4–10 × 10^3^/μL) increased significantly with 2.7 × 10^3^/μl (CI95% 0.6–4.8 × 10^3^/μl; *p* = 0.02) so did PMN (normal value 5.6–17 × 10^3^/μl) with 150% (CI95% 61–288%; *p* < 0.001) during cell infusions. Once the whole cycle was completed, PMN and total white blood count normalized after 7 days. No significant difference due to cell infusions has been observed for monocytes.

#### Hemorrhagic risk

**Fig. 3:**
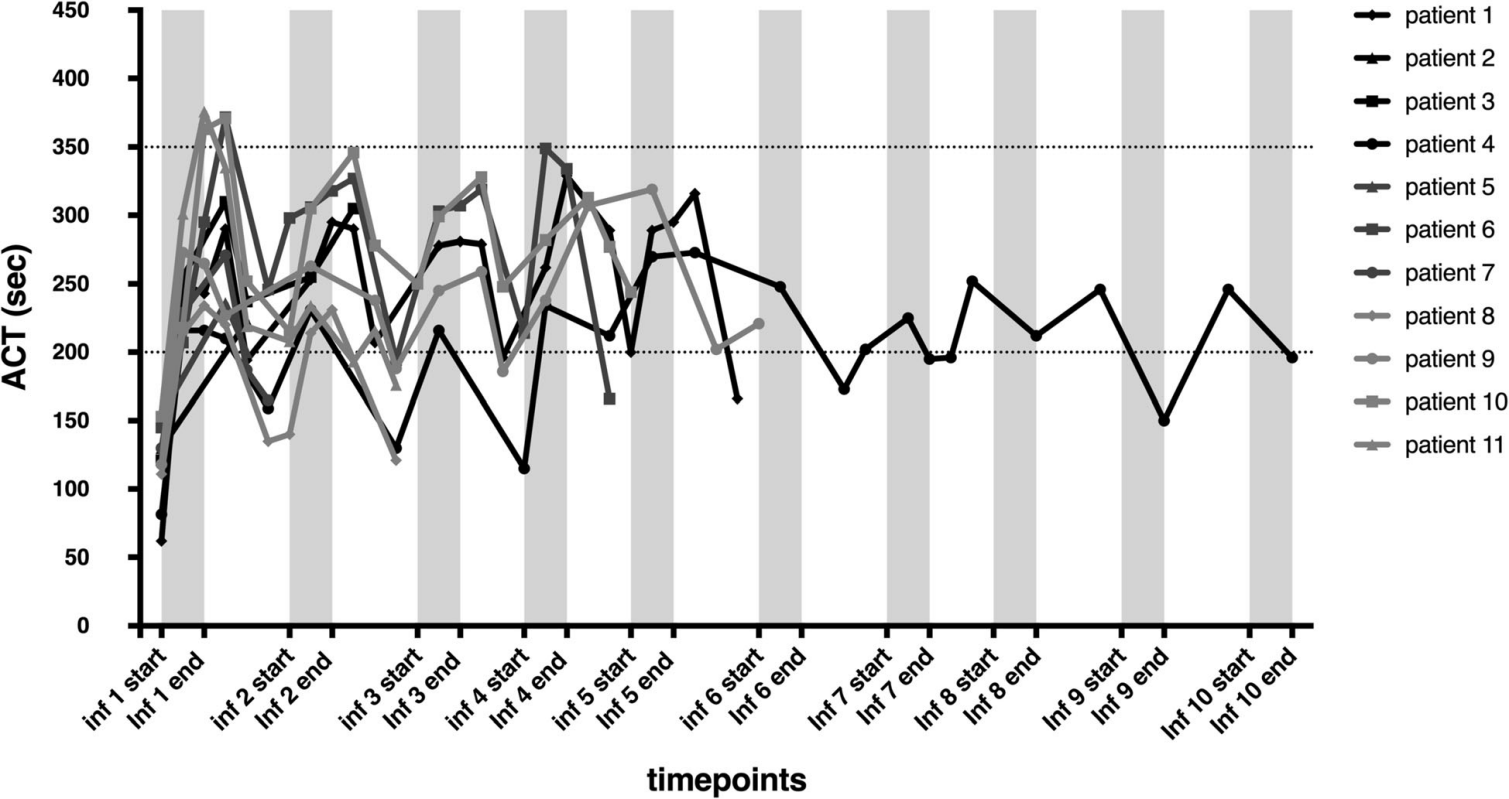
Monitoring of anticoagulation by measuring activated clotting time (ACT) levels during and after intraportal infusion of HepaStem in patients (*n* = 11). ACT levels (targeted values 200–350 s; dotted horizontal lines) are represented for each patient during cell infusions (1–10)

Anticoagulation was monitored by repeated ACT measurements, remaining in the targeted range (200–350 s) for 8 patients (see Fig. 3). In the other 3 patients, bivalirudin administration had to be reduced until ACT levels decreased beneath 350 s. No major adverse effects were caused by the use of anticoagulant treatment. Four patients, including one patient with too elevated ACT levels, presented minor adverse effects. One patient presented petechiae 24 h after the infusion, not associated with thrombopenia. Two patients presented epistaxis, of which one during infusion with high ACT levels, resolving once bivalirudin doses were reduced. Finally, one patient presented a minor hemorrhage at the entrance site of the catheter, resolving without sequelae.

## Discussion

In this phase 1/2 clinical study, patients with urea cycle disorders (UCD) and Crigler-Najjar (CN) syndrome were treated with three different cell doses: low (12.5 × 10^6^ cells/kg), intermediate (50 × 10^6^ cells/kg), and high (200 × 10^6^ cells/kg). This study reports that intraportal HepaStem infusion while using an anticoagulant cocktail, heparin (10 I.U./5 × 10^6^ cells), and bivalirudin (1.75 mg/kg/h) is safe. Nevertheless, minor adverse effects were observed. One patient developed a partial left portal vein thrombosis the second day of infusions, causing a mild increase in portal vein pressure, suggesting that cell infusion induced a thrombus only at the infusion site. Two other patients developed high portal vein pressure. One of them presented high D-dimer levels, causing the interruption of the infusion cycle. D-dimers normalized spontaneously after 7 days in all patients, except for the patient who presented partial portal thrombosis who was treated with curative low molecular weight heparin. Portal vein flow could not be analyzed because this parameter results from a delicate equilibrium, influenced by multiple factors such as intraportal cell infusion rate, ingestion of food, or hydration status. These influencing factors were different for all patients, so no analysis could be performed on portal vein flow. Thrombogenic adverse effects seem to be cell-dose dependent. Even though D-dimer levels increased significantly in all patients, abnormal portal pressure was only present in the high-dose group, except for one patient who presented a left portal thrombus after 4 cell infusions in the intermediate group. Cell infusions caused in all patients a significant decrease in hemoglobin and platelets, but they both normalized after 7 days once the whole cycle was completed. D-dimer levels increased significantly at the end of the infusion cycle but normalized spontaneously 7 days after the whole cycle was completed. This suggests that HepaStem activated the coagulation cascade, with the consumption of platelets and production of fibrin, inducing increased levels of D-dimers. But once the infusing cycle was completed, both parameters normalized spontaneously. Patients with CN or UCD do not express coagulation disorders linked to their disease. Total white blood counts and PMNs significantly increased during cell infusions, in contrary to lymphocytes. Again, all parameters normalized spontaneously after 7 days. No coagulation disorders are described in the literature in patients with CN or UCD.

A phase 1b/2a clinical study by Melmed et al. [20] could confirm this hypothesis. Melmed et al. infused human placenta-derived mesenchymal-like cells to treat moderate to severe Crohn’s disease using different cell dosages (1.5–6–12 × 10^8^ cells, corresponding to 2.5–10 and 20 × 10^6^cells/kg for a patient weighting 60 kg). More side effects including venous thrombosis at infusion site were observed in the higher doses of cells. But even at low cell doses (1 × 10^6^/kg), Wu et al. [19] reported venous thrombosis after intravenous umbilical cord MSC infusions in 2 renal transplanted patients with chronical kidney failure. Both thrombi were distant from the puncture site and associated with an elevation in D-dimer levels. Cell therapy is mainly administered by peripheral intravenous infusions like the two previously cited studies. Johansson et al. [32] nevertheless transplanted by intraportal infusion-isolated human pancreas islets, expressing high levels of tissue factor (TF) like MSCs. All nine patients presented a significant increase in D-dimer levels and in thrombin generation marker thrombin-antithrombin (TAT). TAT levels normalized after 1 day, but D-dimer decreased slower. Others [21] studied whether co-transplantation of ex vivo expanded autologous bone marrow-derived mesenchymal stem cells (MSCs) with islets is safe. Portal vein thrombosis (PVT) was present in 2 out of 3 patients after infusion. Based on historical control patients, they showed that patients presenting PVT were transplanted with islet pellets with a higher average weight. These studies show that the infusion site does not influence the activation of coagulation, but once the TF-bearing cell is in contact with blood coagulation starts. Numerous studies [13, 16, 33, 34] showed that the procoagulant activity (PCA) of MSCs is directly linked with the amount of TF expressed by the cells. Some researchers exploit even the PCA of MSCs and use them to treat hemorrhagic diseases. Moll et al. [13, 33] infused human bone marrow-derived MSC and placenta-derived decidual stromal cells (DSC) using low cell doses (1–3 × 10^6^cells/kg) by peripheral intravenous catheter. The aim was to treat patients with severe complications, such as hemorrhages due to graft-versus-host disease and hemorrhagic cystitis after allogeneic hematopoietic stem cell transplantation. The infusion of BMMSC induced a weak but significant drop in platelet counts, with a fivefold increase in TAT. Nevertheless, other blood markers, such as leukocytes, neutrophils, and D-dimer did not change significantly. DSC infusions on the contrary induced a significant increase in D-dimers, which normalized after 24 h, but no significant change in platelets. A study in 2017 [35], where heparin (60–300 I.U.) was infused before and after DSC infusions, did not show any significant change in platelets, thrombogenic adverse effect, or hemorrhages between the DSC infused and control group. In our study, anticoagulation was monitored during cell infusions with repetitive measurements of ACT. ACT levels stayed in the optimal range for almost all patients, except for 3 of them with rapid normalization of ACT levels by reduction of bivalirudin dosage. No major adverse effects were observed while using the anticoagulant cocktail. No patients hemorrhaged when the percutaneous catheter was removed. One patient, with normal ACT levels, presented a minor hemorrhage at the insertion site of the catheter after the third infusion. This was probably due to the movement of the catheter, which moved out of the portal vein, causing a premature stop of the infusion cycle. Two patients, one with high ACT levels, presented epistaxis that stopped rapidly once bivalirudin therapy was decreased. The other patients presented epistaxis in the week following the infusion that resolved spontaneously, probably due to a pre-existing condition. Finally, one patient presented moderate petechiae, without thrombocytopenia, 24 h after the complete cell cycle, which resolved spontaneously without sequelae. ACT levels stayed in the optimal range for 3 out of 4 patients presenting hemorrhagic events. These minor adverse effects are thus unlikely caused by the use of our anticoagulant drugs.

This study shows that intraportal infusion of HepaStem with the use of an anticoagulant cocktail, bivalirudin, and heparin is safe. The thrombogenic risk caused by these infusions seems to be cell dose-dependent, with a higher risk if a high cell dosage is used. Only minor hemorrhagic adverse effects were observed, and just one seemed related to the use of anticoagulation drugs. Nevertheless, our data suggests that PCA of HepaStem, like other MSCs, still induces activation of coagulation in recipient patients, even if anticoagulation is well controlled. Due to the small number of patients in each group, no statistical analysis could be performed to analyze if cell dosage induces a significant difference in the activation of the coagulation cascade. Further studies with larger cohorts are needed to answer this question.

## Conclusion

Thrombogenic risk induced by MSC infusion is still a concern in cell-based therapy. Our study used a combination of anticoagulant drugs, heparin, and bivalirudin, to control the thrombogenic risk during cell infusions in 11 patients. Even if our data suggests an activation of the coagulation cascade in these patients with anticoagulant drugs, this was spontaneously reversible in time and showed no deleterious effect on hepatic function. We conclude that using heparin and bivalirudin during HepaStem infusion is safe.

## Supplementary information

**Additional file 1.** Consort flow diagram of the studied population.

## Data Availability

The datasets used and/or analyzed during the current study are available from the corresponding author on reasonable request.

## Abbreviations

ACT: Activated clotting time
ALT: Alanine aminotransferase
AST: Aspartate aminotransferase
CN: Crigler-Najjar
DMSO: Dimethyl sulfoxide
DSC: Decidual stromal cells
IBMIR: Instant blood-mediated inflammatory reaction
MSC: Mesenchymal stem cell
OLT: Orthotopic liver transplantation
OTC: Ornithine transcarbamylase
PCA: Procoagulant activity
PMN: Polymorphonuclear
PVT: Portal vein thrombosis
TAT: Thrombin-antithrombin
TF: Tissue factor
UCD: Urea cycle disorder
