# Supplementary material for: Infusion-related thrombogenesis by liver-derived mesenchymal stem cells controlled by anticoagulant drugs in 11 patients with liver-based metabolic disorders
Source: Stem Cell Res Ther. 2020 Feb 7;11:51. doi: 10.1186/s13287-020-1572-7 (PMC7006410; doi:10.1186/s13287-020-1572-7)

CONSORT 2010 Flow Diagram for the paper:

Infusion related thrombogenesis by liver derived mesenchymal stem cells controlled by anticoagulant drugs in 11 patients with liver based metabolic disorders.

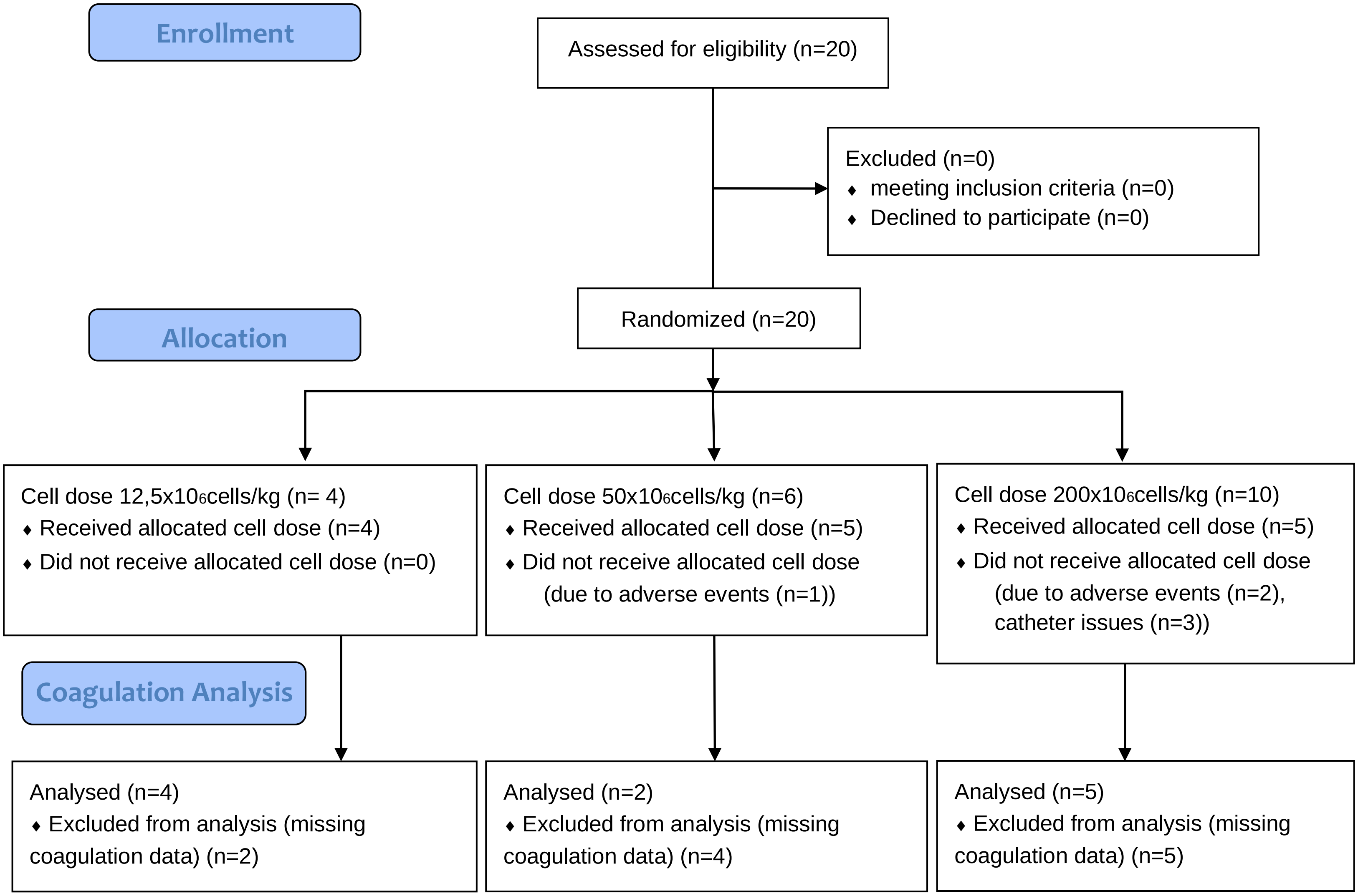

Supplement: Supplementary file 1 — Additional file 1. Consort flow diagram of the studied population. [file 13287_2020_1572_MOESM1_ESM.pdf]
